# The effect of tetrahedron framed permeable weirs on river bed stability in a mountainous area under clear water conditions

**DOI:** 10.1038/s41598-017-04711-8

**Published:** 2017-07-07

**Authors:** Jia-mei Wang, Xing-guo Yang, Hong-wei Zhou, Zi-hao Wang, Jia-wen Zhou, Yu-feng Liang

**Affiliations:** 1 State Key Laboratory of Hydraulics and Mountain River Engineering, Sichuan University, Chengdu, 610065 PR China; 2 College of Water Resource and Hydropower, Sichuan University, Chengdu, 610065 PR China

## Abstract

A flexible riverbed protection called tetrahedron framed permeable weirs (TFPW) is proposed to protect riverbeds in mountainous areas from scouring. Under clear water conditions, a series of laboratory flume experiments were performed to study the effects of TFPW with different layout types on the stability of riverbeds. The objectives of this paper were to advance understanding of the role that TFPW play in the erosion process of river beds and to optimize the TFPW design for reducing velocity, promoting sediment deposition and good structural stability. Data on velocity distribution and variation, equilibrium bathymetry, flow resistance, bed form characteristics and structural stability were collected and analyzed. The results indicate that (1) with good structural stability, all the TFPW with different layout types had significant effects on the stabilization of the riverbed by reducing velocity, raising the water level, increasing the roughness coefficient, protecting the riverbed from degradation and promoting deposition; and (2) the random Double TFPW with large rates of deceleration, large deposition ranges, and good structural stability, and the paved Single TFPW with small rates of deceleration but large deposition ranges and perfect structural stability, were suitable and optimal for riverbed protection in a clear water channel.

## Introduction

Human activities, such as dam/reservoir construction, stream bed mining, and land-use changes, as well as natural processes such as landslide damming, will change the natural flow and sediment regimes of the river^1–7^ and this can lead to general scour in the river^8^. Particular in mountainous areas, increase of the vegetation cover, with the sediments retained behind check dams, reservoirs and dams, result in sediment supply from upstream reduced remarkably, which would lead to clear water conditions in real situations. The degradation of streambeds caused by the clear water scour of mountainous rivers results in riverbank and structural failure of bridges, underwater pipelines, water intakes, invert siphons and other structures. Traditionally, in-stream grade control structures are the common solution for stabilizing riverbeds and riverbanks, and for protecting river crossing structures^9^. Grade control structures (also called drop structures, stabilizers, weirs, barrages, or check dams) are impermeable or solid structures^10^. While a solid grade control structure stabilizes the upstream channel bed, it usually induces downstream local scour, which will pose a threat to the structure itself^11–13^. Thus, each structure needs adequate upstream and downstream protection, such as riprap or gabions^10, 12^. In addition, impermeable structures have another major disadvantage of not being ecologically friendly by remarkably constituting barriers for fish migration^14^. Recently, low-head hydraulic structures, such as block ramps, rock sills, and gabion weirs^15–17^, three-dimensional grade control structures, for example, A-, U- and W-shaped rock weirs^18–21^, and open hydraulic structures, such as Log-vane, J-Hook vane and Cross-vanes^22–24^, have been widely used in river restoration. While they regulate the sediment transport, promote bed stabilization and assure optimal habitat for fish species in the river, they strongly affect river morphology because of the local scour processes occurring downstream of the structures^15–24^. The scour downstream of the grade control structures is a result of jets (free over fall plunging jets, submerged jets, vertical jets, horizontal jets, and impinging jets) over the impermeable grade control structures^11, 12, 25^.

To prevent river bed scouring by jets, tetrahedron frames are proposed for building a permeable weir, or a series of permeable grade control structures known as tetrahedron framed permeable weirs (TFPW). TFPW succeed at considering both hydraulics and ecological conditions. From a hydraulic point of view, the tetrahedron frames permit flow through the TFPW at reduced velocities, thereby preventing bed erosion and causing deposition of sediment from the flow. Their mechanisms are similar to the permeable dikes, such as the timber pile dike and steel jack^9^. However, due to the intense turbulence exerted by the tetrahedron frames, high energy dissipation is produced^26^. The advantages over traditional solid grade control structures are that they will not generate jets, so they rarely cause local scour. Meanwhile, tetrahedron frames are permeable, flexible, and low-environmental impact structures.

Recently in China, since they were produced in 1990 for river improvement, bank protection and flood prevention emergency work, tetrahedron frames have been more and more widely used for river engineering, including the protection of bridge piers, central bars, groin and stilling basins, and even the scour protection downstream from grade control structures^26–30^. For the first time, a tetrahedron framed dam was introduced for channel regulation in the lower reaches of the Yangtze River in 2013. However, tetrahedron frames have not been used for river bed stabilization as TFPW. The interaction between tetrahedron frames and fluid flow has been studied by Lu *et al*. (2011) experimentally^26^. They showed that the tetrahedron frame disturbed the flow fields significantly, resulting in decreasing velocity, Reynolds shear stress, and total shear stress; as well as increasing turbulence intensity. These were the causes of enhancing energy dissipation and reducing the probability of sediment entrainment and even inducing sediment deposition. This finding provided the possibility of TFPW being grade control structures by reducing flow velocity, raising the upstream water level, limiting excessive bed degradation, and promoting bed stabilization. There is a lack of information on important aspects of TFPW.

The aim of this study is to experimentally analyze the effect of TFPW on the stability of river beds in mountainous areas under clear water conditions. In detail, the first objective is to advance understanding of the role that TFPW play in hydraulic and bed form characteristics. The second objective is to optimize TFPW design for reducing velocity, promoting sediment deposition, and having good stability, by conducting a series of experiments with different TFPW layouts and different spacing between two TFPW.

## Methods

### Installation and instruments

**Figure 1:**
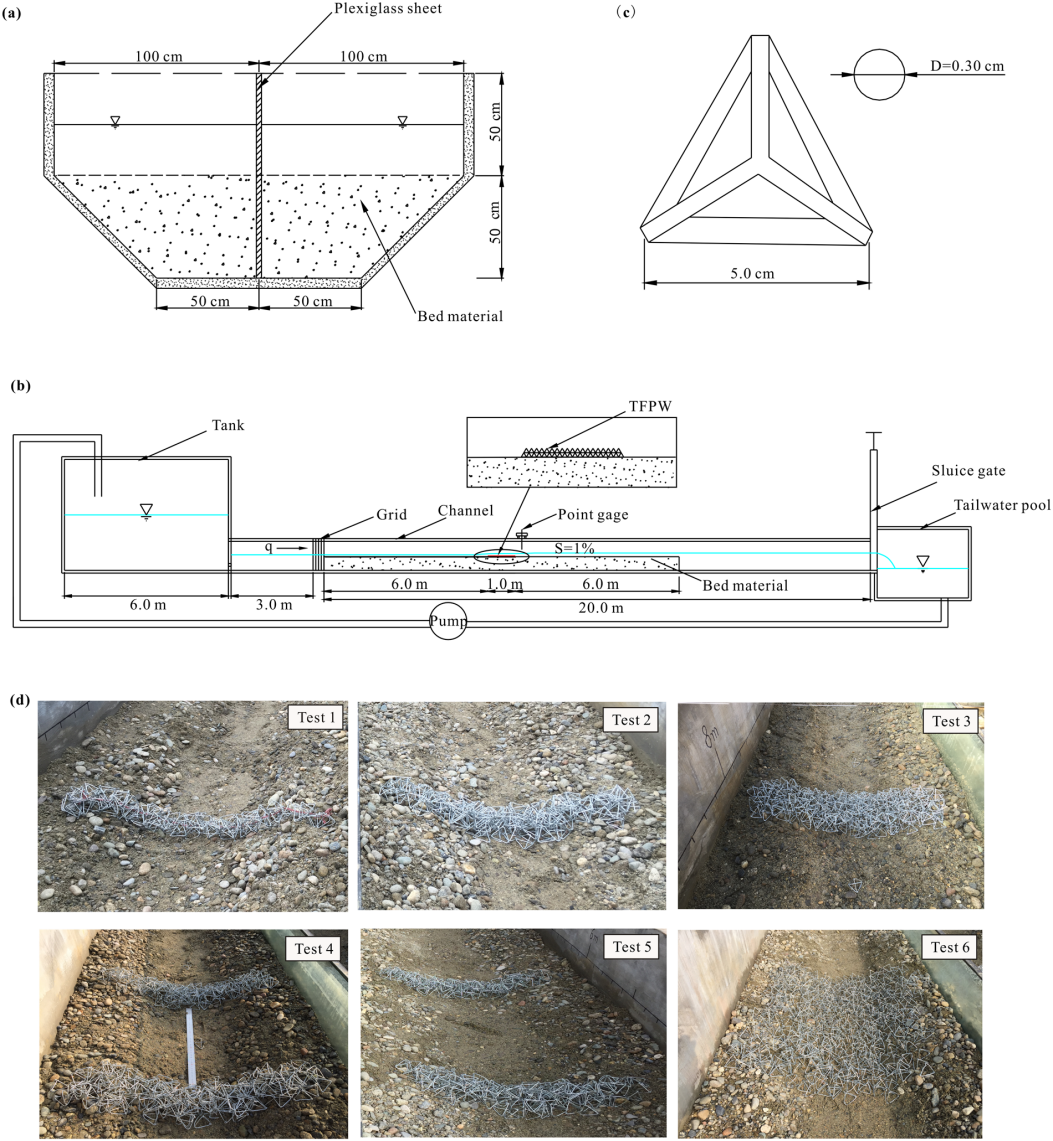
Experimental flume design: (**a**) cross section of the channel; (**b**) definition sketch of the experimental installation; (**c**) sketch of tetrahedron frame model and (**d**) schematic patterns of six TFPW used in this study.

Tests were carried out in two symmetrical flumes under clear water conditions. These two flumes were separated by a Plexiglas sheet, where each experimental flume is the width of 1.0 m, the length of 20.0 m and the depth of 1.0 m. Therefore, two laboratory tests with different TFPW can be carried out at the same time. The cross section of the channel was combined with the upper rectangular section and the lower trapezoid section. The lower section was filled with sediment particles (Fig. 1a). The channel slope was 1%, which represents a typical river bed slope in mountainous regions with a high bed gradient. A tank (1.5 m deep and of surface area 6.0 m × 3.0 m) supplied the approaching stable flow. A tail water pool (1.5 m deep and of surface area 3.0 m × 3.0 m) collected the water, and four diving pumps were used for providing all the experiments with circulating water. Grids for energy dissipation were installed between the tank and the test section of the channel. An adjustable sluice gate at the end of the channel was used to control the tail water depth. A schematic representation of the experimental apparatus is shown in Fig. 1b. The TFPW were placed perpendicular to the flow, whose crests extended across the channel. The choice of the frame models followed the geometric similarity, with a proportional similarity of 1:20. In this study, the tetrahedron frame model consisted of six identical bars, each with a circular cross-section whose diameter was 3 mm, and the length was 5.0 cm (Fig. 1c). A calibrated tank with a precision of ±0.1 L/s was used to measure the discharge flow. To read the water levels and the bathymetry of the mobile bed, a point gauge with reading accuracy of ±0.1 mm was used. The flow velocity was measured by photoelectric propeller current meters along the centerline of the flume both upstream and downstream of the TFPW.

### Bed sediment

To simulate gravel beds with a wide size distribution in mountainous rivers, the bed of the flume was covered by non-uniform sediments that had particle size characteristics with *s* = *ρ*_*s*_/*ρ* = 2.65, *σ* = (*d*_84.1_/*d*_15.9_)^0.5^ = 9.165 and *d*_50_ = 10 mm (where *s* is the relative density of sediment; *σ* is the particle uniformity factor; *ρ*_*s*_ is the density of bed material; *ρ* is the water density; *d*_50_ is the average value of the particle diameter; and *d*_84.1_ and *d*_15.9_ are 84.1%, and 15.9% finer diameters, respectively). At the beginning of each test, the channel bed was carefully paved as natural river morphology. In addition, in order to maintain the same gradation curve, the bed material was mixed after each experiment.

### Experimental scheme

A series of experiments were carried out under clear water conditions. Some included tests on a Single TFPW that was laid out in three different ways, including tied together in series with wire, thrown together randomly, and paved by an orderly layer of tetrahedron frames, which are the common layout types in closure work of the Dujiangyan Irrigation Project, the bank protection and flood prevention emergency work, as well as the central bar protection project, respectively. The others contained the tests on Double TFPW that were thrown together randomly with different spacing (*d*) of 0.0 m, 0.5 m, and 1.0 m, respectively. Six different forms of TFPW were installed in the bed of each flume and the TFPW were tested under the same flow conditions. The TFPW patterns are shown in Fig. 1d. Each experimental test had a duration of between 1.5 and 3.0 hours. The design details of the TFPW layout and the experimental data are shown in Table 1.

**Table 1:** Summary of TFPW dimensions and experimental data for each test.

No.	Structure type	Layout type	Number of frames	*N*	*W* (cm)	*d* (cm)	*h*_*st*_ (cm)	*Q* (L/s)	*S*_*0*_
1	Single TFPW	tied	100	1	10	—	7.50	62.6	0.01
2	Single TFPW	dumped	118	1	10	—	7.50	62.6	0.01
3	Double TFPW	dumped	200	2	10	0	7.50	62.6	0.01
4	Double TFPW	dumped	224	2	10	50	7.50	62.6	0.01
5	Double TFPW	dumped	226	2	10	100	7.50	62.6	0.01
6	Single TFPW	paved	352	1	100	—	3.75	62.6	0.01

Note: *N* = number of weirs; *W* = width of a single structure; *d* = distance between the Double TFPW; *h*_*st*_ = height of the TFPW; *Q* = flow discharge; *S*_0_ = slope of flume. TFPW layouts are shown in Fig. 1d.

### Data collection and processing

The breadth depth ratio of the channel is high (*B*/*h* ≈ 10, where *B* is the width of the flume, and *h* is depth of flow), so the flow field can be treated as two-dimensional open channel flow. After the equilibrium state is reached for each test, the time-averaged velocity at a vertical distance of *y*/*h* = 0.2 from the bed and at the free surface of *y* = *h* were measured both upstream and downstream of the TFPW, where y is the distance in latitude of channel. At the end of every test, the final morphology of the river bed protected by different TFPW was observed and compared with the original bed form before scouring. The deposition lengths both upstream and downstream of the TFPW were measured, as well as the variations in typical cross-sectional profiles before and after scouring by clear water. Further, in an attempt to obtain a more quantitative relationship, sediment samples were obtained in the upper and lower reaches in every test. A sieve analysis of riverbed sediment after scouring was performed, along with the sediment of the original riverbed before scouring. The variations of particle size distribution before and after scouring in the flume with and without TFPW were analyzed. The loss number of tetrahedron frames was counted. Further, by dividing the total of the tetrahedron frames by the amount of lost ones, the loss rate for the tetrahedron frames was calculated. To quantitate the entire stability of the structures, the widths of TFPW, *W*, and its maximum displacement ∆*d* with respect to time were measured. When *W* was increased, it showed that the tetrahedron frames were dispersed by the flow, with a lower total height and a wider width of the TFPW. If ∆*d* increased, two phenomena might occur: one might be that the shape of the weir varied from a straight line to a convex curve toward downstream and another might be a holistic movement of the TFPW.

**Figure 2:**
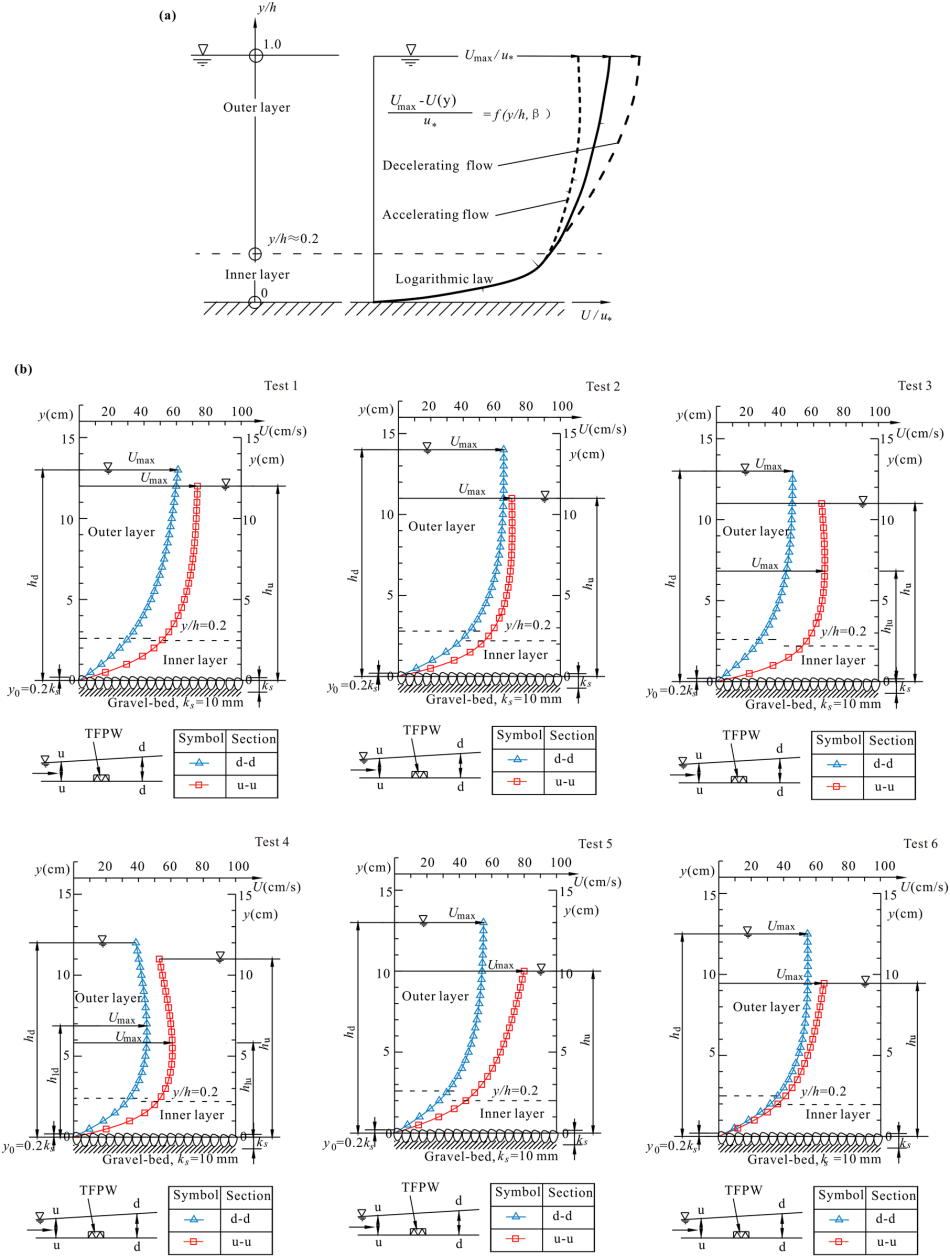
Velocity distribution: (**a**) schematic time-averaged velocity distribution of non-uniform flow (Graf, 1998)^31^ and (**b**) vertical distribution curves of time-averaged velocity for the six different TFPW in this study.

According to Graf^31^, based on a dimensionless parameter *β*, under the non-uniform flow condition, the turbulence in the open channel can be divided into three kinds of flow, including uniform flow, accelerating flow and decelerating flow. Figure 2a shows a schematic time-averaged velocity distribution of non-uniform flow. Furthermore, the mean velocity in the section can be calculated from the velocity distribution. Quantitatively, a parameter *η* called rate of deceleration is used to represent the velocity variation from upstream of the structure to downstream, which can be given in functional form as

1 $$\eta =\frac{{\rm{\Delta }}U}{{U}_{u}}\times 100 \% $$

where ∆*U* = *U*_*u*_ − *U*_*d*_, *U*_*u*_ and *U*_*d*_ are the mean velocity in sections upstream and downstream of the TFPW, respectively.

The TFPW have greater impact on the near-bed flow both upstream and downstream. The turbulent flow fully develops in the inner layer, whose velocity scale is defined by the shear velocity u_*_. The velocity scale u_*_ can be used to nondimensionalize the near-bed velocity *u*, which is more universal. The non-dimensional velocity^32^ *u* ^+^ can be expressed as

2 $${u}^{+}=\frac{u}{{u}_{\ast }}$$

where u is the measured value of near-bed velocity at a height of *y*/*h* = 0.2, and u_*_ is the shear velocity, which can be obtained by bed shear stress τ_0_, and finally expressed by flow depth *h*, bed slope *S*_0_, and acceleration due to gravity *g*. It is as follows:

3 $${u}_{\ast }=\sqrt{gh{S}_{0}}$$

The non-dimensional velocity values were calculated. Similar to the rate of deceleration of mean velocity of section *η*. A parameter *η*′ is used to show the decreasing degree of non-dimensional near-bed velocity under the effects of the TFPW, which is as follows:

4 $$\eta ^{\prime} =\frac{{\rm{\Delta }}{u}^{+}}{{u}_{u}^{+}}\times 100 \% $$

where ∆*u* ^+^ = *u*_*u*_^+^ − *u*_*d*_^+^, *u*_*u*_^+^ and *u*_*d*_^+^ are the non-dimensional near-bed velocity upstream and downstream of the TFPW, respectively.

In each experiment, the equilibrium bathymetry was observed both upstream and downstream of the TFPW. Based on the velocity and water depth, the flow resistance both upstream and downstream of the TFPW was studied. By simplifying the flow in the flume as two-dimensional flow in an open channel, the flow resistance is caused by the surface roughness of the river bed. Traditionally, the flow resistance can be presented by the friction factor *f*, Chezy coefficient *C*, and the Manning roughness coefficient *n* ^33^. The friction factor *f* can be expressed from the Darcy-Weisbach formula, which can be expressed as a function of dynamic pressure due to an average velocity, *U*, of flow. Finally, *f* can be calculated by mean velocity in section *U* and shear velocity u_*_ as follows:

5 $$f=8\frac{{u}_{\ast }^{2}}{{U}^{2}}$$

Combining the Chezy formula with the Darcy-Weisbach formula, the Chezy coefficient *C* can be calculated from the friction factor *f* as

6 $$C=\sqrt{\frac{8g}{f}}$$

Further, using the Chezy formula in the Manning formula, the most widely used roughness coefficient *n* can be given due to Chezy’s *C* as

7 $$n=\frac{1}{C}{R}^{1/6}$$

where *R* is hydraulic radius, *R* = *h* for open channel flow. According to Eq. (5) to Eq. (7), expressed by *f*, *C* and *n*, the flow resistance both upstream and downstream of the TFPW could be calculated. ∆*n* is the increased value of the roughness coefficient *n* from upstream to downstream.

Usually, a threshold velocity is used as a description of the sediment threshold condition. An average velocity of *U*_cr_ and a near-bed velocity of *u*_cr_ at the particle level are both used. In this paper, the equations for hydraulically rough flow put forward by Grade^34^ were used. They were obtained by analyzing a large number of data on the threshold condition, expressed as

8 $${U}_{cr}={({\rm{\Delta }}gd)}^{0.5}(0.5\,\mathrm{log}\,\frac{h}{d}+1.63)$$

9 $${u}_{cr}=1.51{({\rm{\Delta }}gd)}^{0.5}$$

where *h* is the flow depth; and *d* is the representative sediment size, namely, the median diameter *d*_50_, which can be obtained from the particle size distribution curves. ∆ is the submerged relative density (∆ = *s* − 1), and *s* is the relative density of sediment (*s* = *ρ*_*s*_/*ρ*). Based on the measured data of flow depths and 50% finer particle size of the deposition layers both upstream and downstream of the TFPW, the values of the threshold velocities *U*_cr_ and *u*_cr_ were calculated. Therefore, the relationship between velocity and sediment can be further understood.

## Results

### Velocity distribution and variation

The schematic vertical distribution of time-averaged velocity for the six different TFPW is shown in Fig. 2b. For the time-averaged velocity distribution curves both upstream and downstream of the TFPW, the experimental data were in agreement with the log law in the turbulent wall shear layer and the log-wake law in the outer layer. However, for the six TFPW with different layout forms, the flow had different heterogeneity. For the Single TFPW in Test 1 and Test 2, the flow both upstream and downstream of the TFPW was decelerating flow, and the maximum velocity U_max_ points were at the free surface (*y* = *h*) where U_max_ is maximum velocity along vertical direction. Under the influence of the Single TFPW, from upstream to downstream, with the decrease of mean velocity in the section, the velocity distribution along the vertical direction tended to be more uniform. When it came to the Double TFPW with the *d* = 0.0 m in Test 3, which was twice as wide as the Single TFPW in Test 2, the flow upstream of the structure tended to be accelerating flow and the maximum velocity occurred under the free surface where *h*_1_ < *h*, in which h_1_ is flow depth where the maximum velocity occur. After flowing through the TFPW, the flow converted back into decelerating flow, whose maximum velocity point was at the free surface. Similarly, in Test 4, the maximum velocity occurred under the free surface for the Double TFPW with a distance of 0.5 m both upstream and downstream. The distance between the Double TFPW increased to 1.0 m in Test 5; however, the accelerating flow recovered the decelerating flow both upstream and downstream of the structure. The velocity distribution along the vertical direction downstream of the structure tended to be more uniform than that upstream of the structure. In Test 6, where the Single TFPW was tiled by a layer of tetrahedron frames, which was more similar to a broad crest weir, the velocity distribution was similar to Test 5. The flow both upstream and downstream of the TFPW was decelerating flow making it more uniform but without generating severe disturbance. In general, every kind of TFPW had a good effect on the open channel flow by promoting the homogeneity.

With different layout types of tetrahedron frames, under the protection of every kind of TFPW, velocities were reduced significantly. The values of the deceleration rate *η* are shown in Table 2. In particular, for the Single TFPW in Test 1 and the Double TFPW in Tests 3–5, the values of *η* were greater than 26%. However, in Test 2 and Test 6, the values of *η* were slightly smaller. For the Single TFPW with different layout types, the rate of deceleration of mean velocity in the section in Test 1 (*η* = 26.79%) was much larger than that in Test 2 (*η* = 10.60%) and Test 6 (*η* = 5.67%). The Double TFPW with a spacing of *d* = 0.0 m in Test 3 had a much larger rate of deceleration (*η* = 36.91%) than the Single TFPW in Test 2 (*η* = 10.60%). For the Double TFPW in Tests 3–5, with the distance between the two TFPW increasing from 0.0 m to 0.5 m and to 1.0 m, the value of *η* gradually decreased (from 36.91% to 28.50% and to 26.30%).

**Table 2:** Results of the mean velocities in each section and the corresponding rates of deceleration for each test.

No.	*U*_*u*_ (cm/s)	*U*_*d*_ (cm/s)	∆*U* (cm/s)	*η* (%)
1	60.04	43.95	16.08	26.79
2	60.22	53.84	6.39	10.60
3	58.35	36.81	21.54	36.91
4	53.03	37.92	15.11	28.50
5	58.79	43.33	15.46	26.30
6	47.93	45.22	2.72	5.67

Note: *U*_*u*_ = mean velocity in section upstream the TFPW; *U*_*d*_ = mean velocity in section downstream of the TFPW; ∆*U* = difference between the mean velocities in sections upstream and downstream of the TFPW; *η* = deceleration rate of the mean velocities in each section.

The calculations of the deceleration rate of the dimensionless near-bed velocity *η*′ are shown in Table 3. The TFPW had a stronger disturbance to near-bed flow. Compared with the rate of deceleration of the mean velocity in section *η* (Table 2), *η*′ was much larger. The biggest and smallest values of *η*′ were 54.08% and 28.21%, respectively. The change regulation of *η*′ was coincident with *η* for TFPW with different layout types. In other words, for the Single TFPW, the rate of deceleration of near-bed velocity in Test 1 (*η*′ = 46.08%) was larger than those in Test 2 (*η*′ = 33.73%) and in Test 6 (*η*′ = 28.21%). With a broader width, the Double TFPW with a spacing of *d* = 0 in Test 3 had a much higher rate of deceleration (*η*′ = 54.08%) than the Single TFPW in Test 2 (*η*′ = 33.73%). Under the influence of the Double TFPW, the values of *η*′ decreased from 54.08% to 53.32% and to 43.93%, when the spacing increased from 0.0 m to 0.5 m and to 1.0 m.

**Table 3:** Calculations of dimensionless near-bed velocity and the corresponding rates of deceleration for each test.

No	Upstream				Downstream				∆*u* ^+^	*η*′ (%)
	*u*_*u*_ (cm/s)	*h*_*u*_ (cm)	*u*_u*_ (cm/s)	*u*_*u*_^+^	*u*_*d*_ (cm/s)	*h*_*d*_ (cm)	*u*_d*_ (cm/s)	*u*_*d*_^+^		
1	49.00	13.2	11.4	4.310	27.75	14.5	11.9	2.324	1.986	46.08
2	48.26	11.4	10.6	4.571	34.48	13.2	11.4	3.029	1.542	33.73
3	51.43	10.9	10.4	4.969	25.39	12.6	11.1	2.282	2.687	54.08
4	43.21	10.7	10.2	4.222	22.64	13.4	11.5	1.971	2.251	53.32
5	44.73	11.3	10.5	4.268	29.95	16.0	12.5	2.393	1.875	43.93
6	37.23	10.1	9.9	3.754	31.97	14.4	11.9	2.695	1.059	28.21

Note: *u*_*u*_ = measured value of near-bed velocity upstream of the TFPW; *h*_*u*_ = flow depth upstream of the TFPW; *u*_u*_ = shear velocity upstream of the TFPW; *u*_*u*_^+^ = non-dimensional near-bed velocity upstream of the TFPW; *u*_*d*_ = measured value of near-bed velocity downstream  of the TFPW; *h*_*d*_ = flow depth downstream of the TFPW; *u*_d*_ = shear velocity downstream of the TFPW; *u*_*d*_^+^ = non-dimensional near-bed velocity downstream of the TFPW; *η*′ = deceleration rate of dimensionless near-bed velocities.

### Variation in water depth and flow resistance

**Figure 3:**
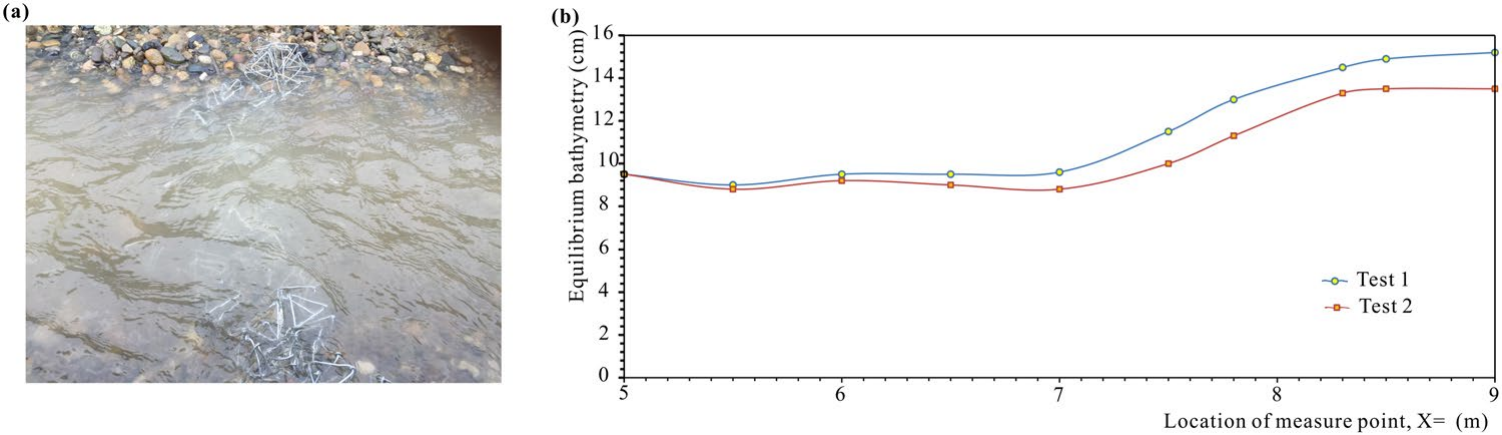
Variation in flow depth: (**a**) photo of the flow surface around the TFPW in Test 1 and (**b**) diagram of depth variation in Test 1 and Test 2, where X is the distance to the inlet of the flume. The TFPW is located at X = 8.0–8.1 m.

Because of the high permeability of the tetrahedron frames, the flow was disturbed slightly while passing through the TFPW, unlike solid structures such as traditional stabilizers, barrages and sills that lead to jets. The submerged TFPW had little impact on the surface of the flow, where the water-depth variation was gentle, as shown in Fig. 3a. The typical variation diagrams of water depth on the central axis of the flume along the direction of the flow are shown in Fig. 3b. Within a certain range upstream of the TFPW, the water depth changed slightly along the flume. The water level remained horizontal or descended slightly. At a distance of approximately 1.0 m upstream from the TFPW (X = 7.0 m), the water level began to rise significantly. When it came to the place within a certain range downstream of the TFPW where X was near 8.3 m, the bathymetry would not increase and the water level tended to be stable. Overall, for all of the six tests, the water levels in the lower reaches were significantly higher than those in the upper reaches. The difference in height of the water surface between upstream and downstream was approximately 2.5 to 4.5 cm. This phenomenon could be explained by hydraulic jump around the TFPW, where the flow regime converted from rapid flow to slow flow, resulting in an especial local hydraulic phenomenon that the water surface suddenly jump. However, the hydraulic jump downstream of the TFPW was much weaker than that downstream of some sluice structures, few local scour would occur downstream of the TFPW. This phenomenon showed that the TFPW would block the water from flowing and cause rising water levels, and a retardation zone existed around the TFPW, even though they were permeable.

The results of the friction factor *f*, Chezy coefficient *C*, and Manning roughness coefficient *n* are shown in Table 4. Obviously, the roughness coefficient *n* upstream of structure was approximately 0.040 for every test, while the maximum was approximately 0.070 downstream of the structures for Test 3 to Test 5. It was because of the influence of the tetrahedron frames that the roughness increased. ∆*n* was the smallest for the Single TFPW in Test 2, at only 0.009. In addition, for the Double TFPW in Test 3–5, ∆*n* was approximately 0.030. Their increasing roughness functions were much better than other TFPW with other kinds of layout types.

**Table 4:** Calculation results of flow resistance including *f*, *C*, and *n* for each test.

No	*f*		*C* (m^0.5^/s)		*n*		∆*n*
	Upstream	Downstream	Upstream	Downstream	Upstream	Downstream	
1	0.288	0.586	16.49	11.57	0.043	0.063	0.020
2	0.248	0.359	17.79	14.79	0.039	0.048	0.009
3	0.254	0.727	17.57	10.39	0.039	0.068	0.029
4	0.296	0.736	16.28	10.33	0.042	0.069	0.027
5	0.255	0.666	17.54	10.86	0.040	0.068	0.028
6	0.341	0.554	15.17	11.90	0.045	0.061	0.016

Note: *f* = friction factor; *C* = Chezy coefficient; *n* = Manning roughness coefficient; ∆*n* = increased value of the roughness coefficient *n* from upstream to downstream.

### Bed form characteristics

**Figure 4:**
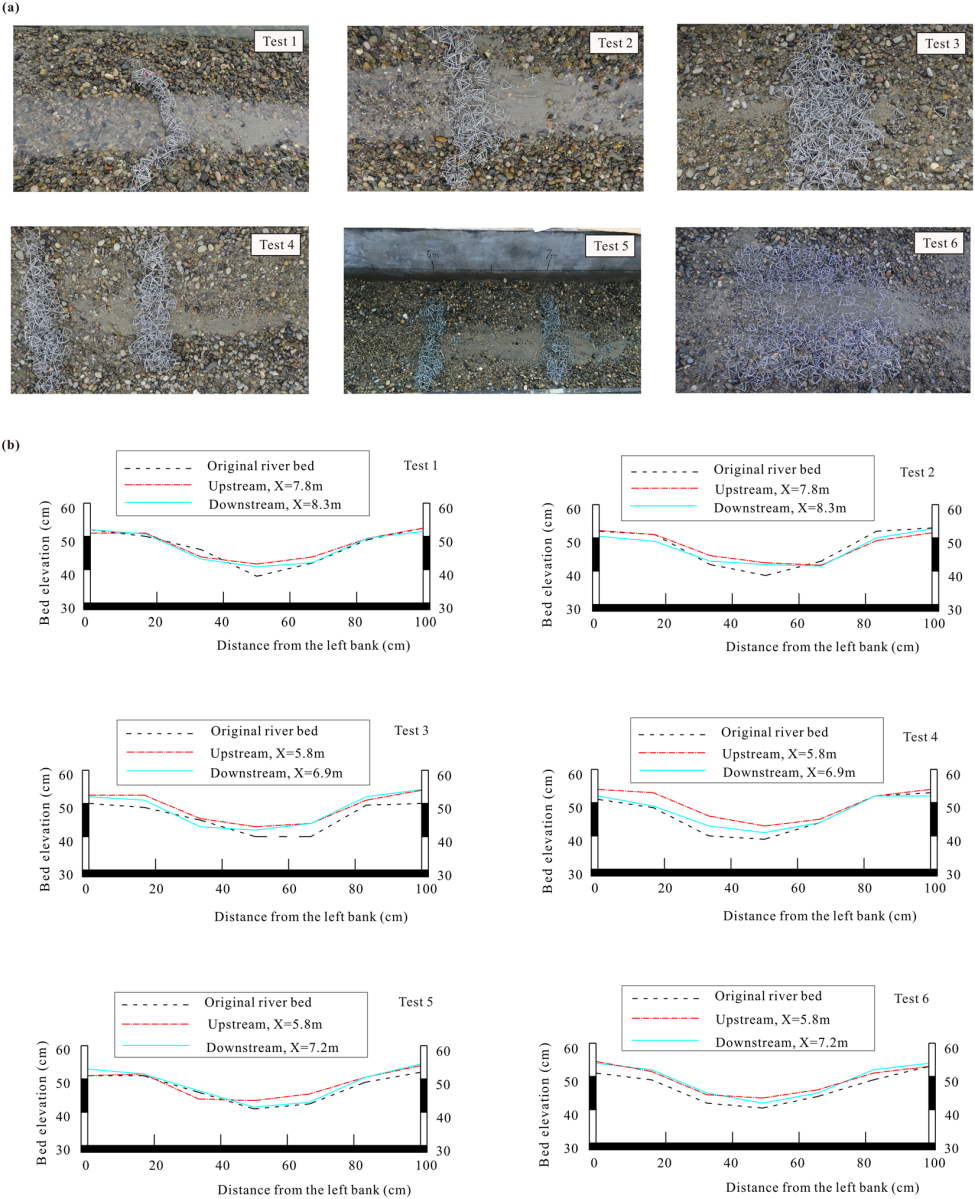
Deposition phenomena: (**a**) photos of the final morphology of a river bed protected by different TFPW and (**b**) variation in typical cross-sectional profiles.

Figure 4a shows the pictures of the final morphology of a river bed protected by different TFPW. Obviously, under the protection of TFPW, an armoring layer existed on the surface of the river bed near the banks after scouring. Whereas in the central channel, a certain range of sediment deposition existed not only upstream of the TFPW but also downstream from them. Interestingly, the particle size of the depositing layers and the dune heights were various from upstream to downstream. It was observed that the dune heights upstream of the TFPW were slightly higher than those downstream of the TFPW and the particle sizes both upstream and downstream of the TFPW were finer than the sediment of the riverbed before scouring. Further, the sediment size of the TFPW downstream deposition was much finer than upstream of the TFPW.

For the TFPW with different layout types, the range of deposition was not the same. In Test 1, although the refinement of the deposition layer was not obvious around the TFPW, it could be observed that the sediments upstream of the Single TFPW were coarser than those downstream of the TFPW. However, the finer particles downstream of the TFPW did not deposit at the heel of the TFPW but were located at a distance of 0.3 ~ 0.4 m from the heel of the TFPW. The Single TFPW in Test 2 had a better effect on promoting sediment deposition than that in Test 1, where the phenomenon of deposition layer refinement both upstream and downstream of the TFPW was more obvious. The finer deposition layer extended from the heel of the TFPW to a certain distance of the lower reach. The deposition layer upstream of the TFPW in Test 3 was higher. At the same time, more particles were deposited within the tetrahedron frames. In Test 4 and Test 5, respectively, the Double TFPW with a spacing of d = 0.5 m and d = 1.0 m had a larger area covered with the deposition layer. It was found that the bed load was deposited not only upstream and downstream of the TFPW but also between the two TFPW. For the Single TFPW paved by an orderly layer of tetrahedron frames in Test 6, the particles deposited within the tetrahedron frames in the upper reach were coarser than those in the lower reach. The final measured results of the deposition lengths both upstream and downstream of the TFPW are shown in Table 5.

**Table 5:** Measured results of the deposition range for each test.

No	*W*_*t*_ (m)	*d* (m)	*l*_*du*_ (m)	*l*_*dd*_ (m)	*l*_*dt*_ (m)
1	0.10	—	0.35	0.30	0.75
2	0.10	—	0.45	0.50	1.05
3	0.20	0	0.50	0.40	1.10
4	0.20	0.50	0.40	0.45	1.55
5	0.20	1.00	0.40	0.30	1.90
6	1.00	—	0.30	0.15	1.45

Note: *W*_*t*_ = total width of TFPW; *d* = distance between the Double TFPW; *l*_*du*_ = deposition length upstream of TFPW; *l*_*dd*_ = deposition length downstream of TFPW; *l*_*dt*_ = total length of deposition.

Figure 4b shows the variation in typical cross-sectional profiles before and after scouring. The erosion characteristics and protecting effects can be briefly summarized as follows: Except in Test 5, the effect of promoting particle deposition was limited. The rise of bed elevation only upstream of the structure was significant, where the bed elevation downstream of the structure was almost the same as the original river bed before scouring. In other tests, under the protection of tetrahedron frames, the bed elevations both upstream and downstream of the TFPW after scouring were much higher than those of the original river bed. In other words, particles were deposited around the TFPW. Further, the bed elevations upstream of the TFPW were slightly higher than those downstream of the TFPW. The TFPW could uplift the bed elevation by promoting sediment accumulation, in addition to the effects on protecting the bed from scouring and degradation.

**Figure 5:**
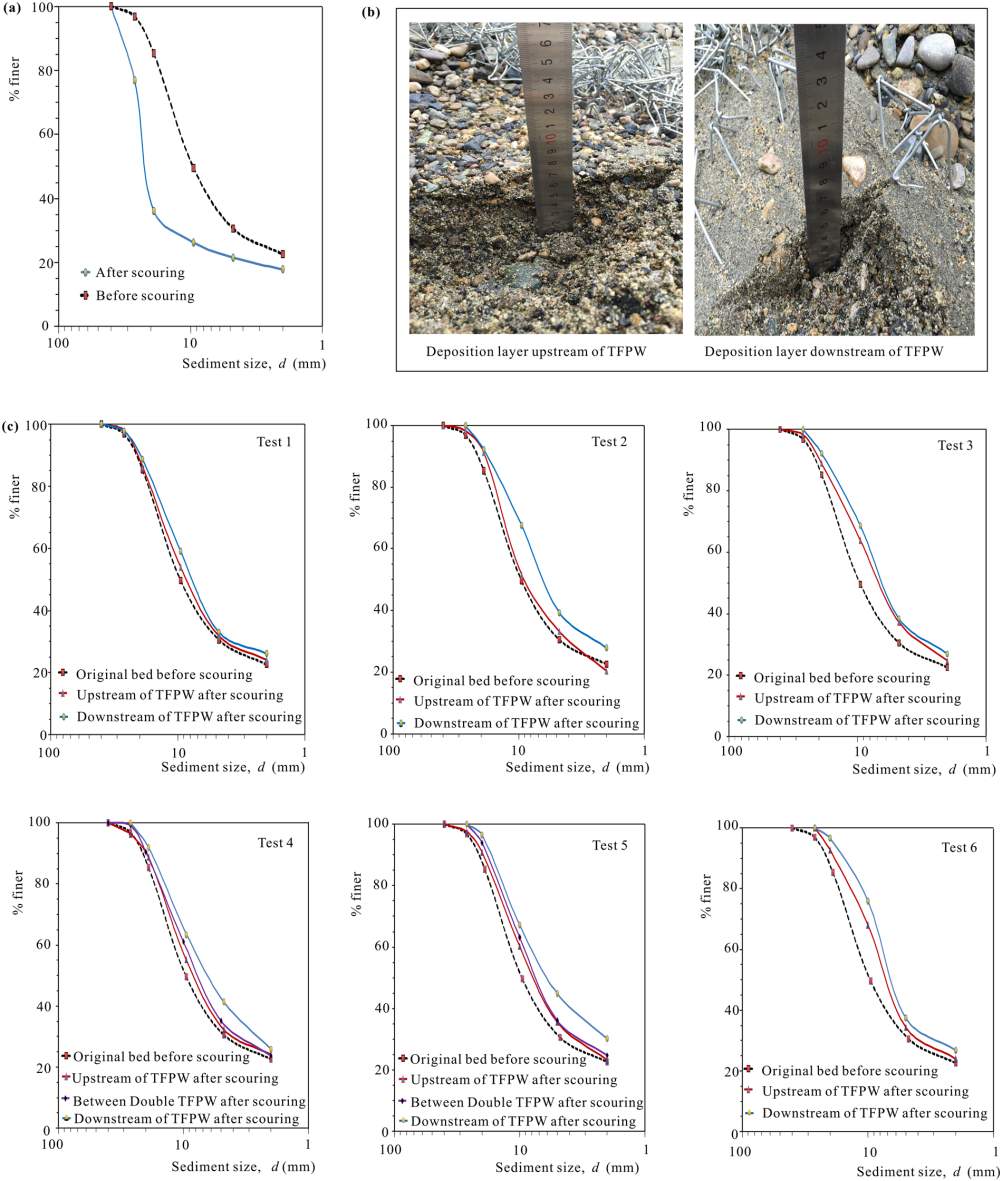
Variation of bed material gradation composition: (**a**) particle size distribution curve of bed materials without any bed protection structures; (**b**) photos of typical deposition layers around TFPW in Test 2 and (**c**) particle size distribution curves of deposition layers under the protection of different TFPW.

Figure 5a shows the variation of particle size distribution before and after scouring by clear water in the flume without any bed protection. According to the particle size distribution curves, the median particle diameter of bed materials increased from *d*_50_ ≈ 10 mm before scouring to *d*_50_ ≈ 25 mm after scouring. Significant armoring phenomenon occurred under clear water conditions. However, under the protection of the TFPW, sediments were deposited, and as shown in Fig. 4a, the grain size characteristics of sediments upstream of TFPW were obviously different from those downstream of the TFPW. Figure 5b shows the details of the deposition layers both upstream and downstream of the TFPW in Test 2. Obviously, the particles downstream of the TFPW are much finer than those in the upper reach. The particle size distribution curves are given in Fig. 5c. The results show that for not only the finer deposition layers downstream of the TFPW and between the double TFPW but also those coarser particles upstream of the TFPW, the riverbed sediments after scouring were finer than those of original bed before scouring. The trend from upstream to downstream was that the sediments tended to be increasingly finer.

### Stability of structures

Generally, the loss rate of tetrahedron frames was low in each test. For the Single TFPW in Test 1, the tetrahedron frames were not lost when bound with wires, and the loss rate was 0. For the Single TFPW in Test 6, the loss rate of the tetrahedron frames was only 1.1%, which is considerably lower than Tests 2–5. The tetrahedron frames thrown together randomly in Tests 2–5 had a higher loss rate average of approximately 10%, and they were 11.9%, 9.5%, 9.4%, and 12.4% for Tests 2, 3, 4, and 5, respectively.

Take the TFPW in Tests 1, 2, and 6 as examples, representing the tetrahedron frame layout in three different ways. According to the experimental phenomena and recorded data, the change of *W* and ∆*d* was an irreversible process. However, the shape changes were controllable and overall steady states were reached during the scouring process. For *W*, it did not change in Test 1 and it increased slightly in Test 6 from 100 cm to 105 cm. In Test 2, however, *W* became two times as wide as before scouring, from 10 cm to 20 cm, reflecting that the frames in Test 2 were significantly dispersed by flow. ∆*d* obviously increased for the Single TFPW in Test 1, where ∆*d* = 25 cm, revealing that the total stability was poor. Both the variation of the TFPW shape to convex toward downstream (Fig. 4a) and a holistic movement of tetrahedron frames were observed during the scouring process. For Test 2, the shape of the TFPW varied from a straight line to a curve, but only a small deformation occurred where ∆*d* = 6 cm. In Test 6, with the smallest displacement of ∆*d* = 3 cm, the TFPW had barely moved (Fig. 4a), showing good stability.

## Discussion

All of the six different kinds of layout forms for the TFPW had a significant effect on the velocity distribution and variation both upstream and downstream of the structures by promoting flow homogeneity and reducing velocity. However, the effect was not the same for different TFPW. The flow both upstream and downstream of the TFPW was almost a decelerating flow, except for the TFPW in Test 3 and Test 4, where an accelerating flow appeared. In Test 3, the cause of the maximum velocity occurring under the free surface was that the flow became three-dimensional under the function of a wider TFPW, which might result in secondary flow. Similarly, the Double TFPW with a distance of 0.5 m had a greater effect on the flow, disturbing the flow both upstream and downstream of the structure where the maximum velocities occurred under the free surface. As the distance between the Double TFPW increased to 1.0 m, the combined effects of the two TFPW on flow did not increase. In contrast, the disturbance was observably reduced where the flow was a decelerating flow.

In Test 1, the tetrahedron frames were tied together in a series with wire, so the frames were densely piled one above another, resulting in an obvious deceleration. During the experiments in Test 2 it was observed that the tetrahedron frames thrown together randomly were washed away or embedded in the river bed more easily. As a result, the frames were not as dense as those in Test 1 and caused fewer disturbances. In Test 6, the TFPW were only paved by a layer of tetrahedron frames, so the structure had a much lower height and had a smaller effect on disturbing the flow. Thus, the value of *η* in Test 6 is much smaller than that in other tests. For the TFPW that were thrown together randomly with a wider breadth, the effects of the tetrahedron frames on flow became larger and the turbulence effects were much stronger, thereby reducing the velocity. For the Double TFPW in Tests 3–5, with the distance between the two TFPW increasing, the combined effects of the double TFPW on disturbing the flow gradually weakened. Therefore, the rates of deceleration *η* and *η*′ were both reduced with the increase of spacing.

The work of Lu *et al*. (2011) also revealed the mean velocity of flow around a tetrahedron frame in a smooth open channel^26^. Results indicated that significant flow reduction occurred under the retardation effect of a tetrahedron frame, especially in the near-bed region. However, the deceleration rates were a little larger than that in our study. The deceleration rate ranged from about 0.45 to 0.6 under the submerged conditions investigated in the study of Lu et al (2011), while the deceleration rates of the mean velocities ranged from 5.67% to 36.91%, and even the deceleration rates of dimensionless near-bed velocity ranged from 28.21% to 54.08% in our study. The difference might be caused by different experiment conditions, especially the bed conditions and the number and layout of tetrahedron frame(s).

In natural rivers without any bed and bank protection engineering, clear water discharging from upstream will cause significant erosion, resulting in channel degradation and bank erosion. For mountainous rivers whose banks are bedrock, river incision is the main erosion phenomenon^35^. The armoring phenomenon of the bed materials with wide size-distribution usually occurs in natural rivers scoured by clear water^36^. However, compared with the bed form of the rivers that are not protected by any structures, the bed form under the protection of TFPW changed greatly. The TFPW had a remarkable protective effect from scouring on the river bed. Under the influence of the TFPW, the sediments became remarkably finer along the stream direction. On one hand, the concentrated array of tetrahedron frames will retard flow and raise the water level, resulting in reduction of flow velocity. Therefore, the bed load with a larger grain size will stop transporting and deposit upstream of the TFPW, whereas the sediment with a finer particle size will transport downstream along with the flow. On the other hand, the effect of tetrahedron frames on disturbing the flow field and increasing the roughness, decreases the velocity further. The deposition of the sediment with a finer particle diameter occurs within a certain range downstream of the structures.

The experimental results indicate that the TFPW with different layout types had different effects on the promotion of sediment deposition. The TFPW in Test 2 with a layout type of tetrahedron frames thrown together randomly had a better effect on promotion of sediment deposition than the TFPW tied together in series with wire in Test 1. As a weir twice as wide as the Single TFPW in Test 2, the Double TFPW in Test 3 had a stronger effect on blocking sediment transportation through the TFPW. Therefore, the deposition layer upstream from the TFPW in Test 3 was higher. The Double TFPW had a superimposed effect on sediment accumulation, meaning the particles were deposited in a larger range, including both upstream and downstream of the two TFPW, as well as the total area between them. The Single TFPW paved by an orderly layer of tetrahedron frames in Test 6 had the most significant effect on promoting bed load deposition. The finer particles especially accumulated within the total range paved by the tetrahedron frames. Similarly, the particles in the upper reach were coarser than those in the lower reach.

With an increase in flow velocity to the sediment threshold value, the sediment particles on the bed surface might start to move. In contrast, when the velocity is reduced to a critical condition, the sediment entrainment might stop, resulting in sediment accumulation on the bed. The calculated values of threshold velocities *U*_cr_ and *u*_cr_ are shown in Table 6. Compared with the measured values of mean velocity from section *U* and the near-bed velocities *u* in Table 2 and Table 3, respectively, *U*_cr_ and *u*_cr_ were much larger. Therefore, the sediment particles were deposited both upstream and downstream of the bed protection structures. Furthermore, the threshold velocity upstream of the TFPW was larger than that downstream of the TFPW. Therefore, the sediment in the upper reach had a coarser particle size than in the lower reach.

**Table 6:** Values of threshold velocities (*U*_*cr*_ and *u*_*cr*_).

No.	*U*_*cr*_ (cm/s)		*u*_*cr*_ (cm/s)	
	Upstream	Downstream	Upstream	Downstream
1	63.24	62.45	56.01	54.34
2	64.66	57.59	58.59	48.22
3	58.17	57.75	50.83	48.98
4	61.99	56.52	55.35	48.22
5	60.40	56.03	54.34	47.06
6	57.06	56.46	50.83	47.84

Note: *U*_*cr*_ = threshold average velocity; *u*_*cr*_ = threshold near-bed velocity.

Consisting of six prefabricated concrete bars, a tetrahedron frame has high water permeability. A small hydrodynamic force is exerted on the frames. Additionally, with a slight weight and low center of gravity, a tetrahedron frame has good stability. A large number of frames stacked together, will interrelate, influence and restrict each other. Therefore, the TFPW have good overall stability. Moreover, deposited sediments will bury the bottom of the frames improving the foundation stability. Nevertheless, under the action of large floods, the loss of washed away tetrahedron frames still occurs, as well as the deformation of the entire TFPW. The stability of the structures was closely related to the layout types of TFPW. The tetrahedron frames in Test 1 would not lose at all. Relatively, the tetrahedron frames thrown together randomly in Tests 2–5 had a higher loss rate of approximately 10%. The tetrahedron frames located at the bottom and the inside of the TFPW had good stability because of restriction by other frames. However, due to randomness and the irregularity of the structural arrangement, the tetrahedron frames located on the surface and at the edge of the TFPW had relatively poor stability, and hardly hampered by other frames, they were easily washed away by flow. In Test 6, the tetrahedron frames spread out on the riverbed had good self-adaptability to the river-bed deformation. Furthermore, the stability of the frames was improved by a large amount of sediment that was deposited within the tetrahedron frames. The TFPW thrown together randomly had better stability from the whole deformation than that tied together in series with wire, but slight deformation occurred. The TFPW that was paved by an orderly layer of tetrahedron frames had the best stability, and it was almost immovable after scouring.

Unavoidably, some disadvantages would appear after long period of TFPW persistence or under extreme flood conditions. The bed-load would deposit around the TFPW and cover the substrate, which would promote the structural stability. However, after accumulation for long period, the coarse sediments would silt the permeable weirs. In particular, some large boulders will deposit in front of the weirs. Accordingly, the functionality of this system would decrease. On the other hand, the bed-load movement would abrase any concrete structures, especially when there are coarse bed materials, such as gravels, cobbles, and even boulders during big floods. And the rods of tetrahedron frame would also be hit by boulders. However, even if the both cases happen, it will be easy to repair the TFPW by moving away the big boulders during low water period, and throwing more frames in the same site or constructing another TFPW downstream of the former site.

## Conclusions

To advance understanding of the engineering effects of TFPW on riverbed protection, a series of experiments on TFPW with six different layout types was carried out in laboratory flumes under clear water conditions. Experimental results of the hydraulic characteristics (including velocity, bathymetry and flow resistance), bed form characteristics both upstream and downstream of different TFPW, and the stability of the structures were presented. The experimental results show that, the TFPW were flexible riverbed protection structures, which not only had high permeability but also retarded flow, in addition to having good self-adaptability to the river-bed deformation. Generally, with good structural stability, all the TFPW with different layout types had a significant effect on the stabilization of the riverbed, including the reduction of velocity, the rise of water level, the increase of the roughness coefficient, protection of the bed from degradation and promotion of sediment deposition.

The Single TFPW tied together in series with wire had a strong interaction with flow but poor adaptability to river-bed deformation. The effect on reducing velocity was perfect, with high deceleration rates of both the mean velocity in each section and the near-bed velocity, *η* = 26.79% and *η*′ = 46.08%, respectively. However, the effect on promoting deposition was relatively poor. Nevertheless, overall deformation and displacement of the structure occurred easily, resulting in poor global stability. The Double TFPW thrown together randomly, disturbed the flow intensively, causing high deceleration rates of *η* over 26% and *η*′ over 43%, and an increase in values of the roughness coefficient ∆*n* to over 0.030. With the increase of width of the Single TFPW, the rate of deceleration and deposition range increased. With the increase of spacing between the Double TFPW, the deposition range still increased, but the rate of deceleration was reduced. This kind of TFPW had good structural stability. Paved by an orderly layer of tetrahedron frames, the Single TFPW disturbs the flow slightly because of its low height. Though the rates of deceleration were smallest, *η* = 5.67% and *η*′ = 28.21%, respectively, the effect on promoting deposition was good. The structure could adapt to river-bed deformation perfectly, resulting in the least loss of frames and smallest deformation and displacement, so that its structural stability was best.
